# A conserved karyotype? Chromosomal rearrangements in *Charadrius collaris* detected by BAC-FISH

**DOI:** 10.1371/journal.pone.0280164

**Published:** 2023-01-11

**Authors:** Paulo Victor de Moraes Ferreira, Talita Fernanda Augusto Ribas, Darren K. Griffin, Luyann André Rodrigues Correa, Melquizedec Luiz Silva Pinheiro, Lucas G. Kiazim, Rebecca E. O’Connor, Cleusa Yoshiko Nagamachi, Julio Cesar Pieczarka

**Affiliations:** 1 Laboratório de Citogenética, Centro de Estudos Avançados da Biodiversidade, ICB, Universidade Federal do Pará, PCT-Guamá, Belém, Pará, Brazil; 2 School of Biosciences, University of Kent, Canterbury, United Kingdom; Virginia Polytechnic Institute and State University, UNITED STATES

## Abstract

Charadriidae comprise 142 valid species and the most recent checklist for the occurrence of this family in Brazil describes 11 species. There are few chromosomal studies in Charadriidae, most of them using a conventional approach. In *Charadrius*, only five species had their karyotypes described by classical cytogenetics, of which four have 2n = 76 (*C*. *hiaticula*, *C*. *dubius*, *C*. *vociferou* and *C*. *collaris*) and one 2n = 78 (*C*. *alexandrinus alexandrinus*). Among these species, only *Charadrius collaris* had the karyotype studied by chromosome painting, which allowed the identification of chromosomal homeologies with the karyotypes of *Gallus gallus* (GGA) and *Burhinus oedicnemus* (BOE). According to the literature, studies performed with BAC-FISH using probes from *Gallus gallus* and *Taeniopygia guttata* (TGU) libraries have shown interactions between macro and microchromosomes and micro inversions in chromosomes previously considered conserved. Other studies have shown the fusion of several microchromosomes, forming new macrochromosomes, leading to a decrease in the 2n of some species. The present study aims to deepen the chromosomal information in *Charadrius collaris* through the application of BAC-FISH with probes from the GGA and TGU libraries, in order to investigate possible rearrangements within the apparently conserved karyotype of this species, and thus better clarify the evolutionary history of the species. *Charadrius collaris* presented 2n = 76 and fundamental number (FN) equal to 94. Comparative mapping of BAC probes from GGA and TGU in *Charadrius collaris* revealed hybridization signals from 26 macrochromosome probes. Probes from microchromosomes 9 to 28 of GGA were also used and revealed 31 hybridization signals. The karyotype is well conserved, but it contains a paracentric and a pericentric inversion on the CCO1 chromosome, a paracentric and a pericentric inversion on the CCO4 and the separation of GGA4 into CCO4 and CCO8, demonstrating that the BAC-FISH approach allows for greater data resolution. More studies are needed to improve the understanding of chromosomal evolution within the order Charadriiformes and thus clarify whether these characteristics demonstrated here are specific traits for *Charadrius collaris* or if other species share these characteristics.

## Introduction

Birds are an extremely successful and highly diverse class of vertebrates, with ~11,000 validated species [1], being the second largest group of vertebrates in Tetrapoda [2]. Most species have a karyotype of around 80 chromosomes (10 pairs of macrochromosomes and 30 pairs of microchromosomes). This chromosomal structure is highly conserved among birds, being found in species of all 40 extant orders [2–4], having undergone few changes throughout evolutionary history [3,5]. Studying chromosome morphology is essential to understand the evolution of the class over millions of years, but this type of study in birds is more challenging than in mammals, because of the presence of numerous microchromosomes. Most avian studies are therefore limited to the analysis of macrochromosomes [3].

Even with a very conserved avian karyotype, the occurrence of rearrangements is mentioned by several studies in the literature, especially in old publications demonstrating differences between karyotypes of different species. Recent publications, however, use more sophisticated analysis techniques, showing the rearrangements in much more detail, as is the case of species of the genus *Salator* (Thraupidae, Passeriformes), where the fission of chromosomes homeologues to pairs 1 from *Gallus gallus* (GGA1) and GGA5 were described, resulting in the formation of four chromosome pairs [6]. A similar situation was found on the genus *Turdus* (Turdidae, Passeriformes) in relation to chromosomal fission in the homeologous to GGA1, followed by a series of inversions (one pericentric and two paracentric) in the fragment corresponding to GGA1q [7]. The reciprocal hybridization of whole chromosome probes from *Gallus gallus* and *Burhinus oedicnemus* (BOE) demonstrated the considerable difference between the karyotype of these two species, due to many chromosomal fusions in the BOE karyotype in relation to the GGA [8]. Using BAC probes from *Gallus gallus* and *Taeniopygia guttata*, it was possible to detect multiple chromosomal fissions in the homeologues of GGA1, GGA2 and GGA4, in addition to the fusion between the chromosome corresponding to GGA6 with the microchromosome GGA17 in the karyotype of *Willisornis vidua* [9].

There are orders in the Class Aves, such as Falconiformes and Accipitriformes, which are often objects of cytogenetic studies because they present a large variation in the diploid number (2n), with some species 2n = ~80 and others with 2n = 40–42, this being the determining factor in the choice of these birds for karyotypic study [10,11]. Charadriidae (Order Charadriiformes), on the other hand, present karyotypes with a much smaller variation between species and a chromosome number close to the ancestral karyotype proposed for birds, with 2n varying between 72–78 chromosomes in most species, except *Vanellus spinosus duvaucelii* which presents 2n = 58 [12–14].

The Family Charadriidae is composed by 142 valid species [15,16] and includes the plovers, lapwings and dotterels. The most recent checklist for the occurrence of Charadriidae in Brazil describes 11 species [17], most of which are found in coastal regions, close to lakes, mangroves and beaches [15]. There are few chromosomal studies in this family, most of which have used a classical approach. In the genus *Charadrius*, only five species had their karyotypes described, of which four (*C*. *hiaticula*, *C*. *dubius*, *C*. *vociferus* and *C*. *collaris*) show 2n = 76 and one (*C*. *alexandrinus alexandrinus*) shows 2n = 78; only *C*. *collaris* occurs in Brazil [8,11]. Among these species, *C*. *collaris* had the karyotype studied by chromosome painting, with results demonstrating a high degree of homeology between the macrochromosomes of *Gallus gallus* (GGA) and *Burhinus oedicnemus* (BOE) [18]. It was described for *Vanellus chilensis* through chromosome painting with GGA whole chromosome probes that the fusion between the homeologues GGA7 and GGA8 would be a common feature within the Charadrii clade [19], being confirmed for this species [18] and also found in *Charadrius collaris* [18] and *Burhinus oedicnemus* [8]. In the suborder Scolopaci, several rearrangements occurred, including the fissions of chromosome pairs 2, 3, 4 and 5 [20] of the avian putative ancestral karyotype (PAK) [3]. In the Scolopacidae family, the fission of PAK1 is also observed [20], while in the Jacanidae several fusions have occurred [21].

Chromosomal painting studies, by allowing the detection of chromosomal homeologies, have brought a great advance in the analysis of karyotypic diversity in birds. These studies made it possible to detect fusion/fission rearrangements and inversions, mainly involving macrochromosomes [22]. Studies performed with BAC-FISH using probes from the GGA and TGU libraries have demonstrated interactions between macro and microchromosomes and micro inversions in chromosomes previously considered conserved [23,24]. Other studies have shown the fusion of several microchromosomes, forming new macrochromosomes, leading to a decrease in the 2n of some species [9,10,25].

BAC-FISH is a combination of two pre-existing techniques, BAC (Bacterial Artificial Chromosome) and FISH (Fluorescence *In-Situ* Hybridisation). Initially, BACs were used to create genomic libraries [26], but soon the possibility of using them as probes for hybridization in metaphase chromosomes was noticed. BACs are used as vectors capable of inserting sequences from 100–150 kb into bacteria [27]. FISH is an established technique in chromosomal studies, which has been used in multiple publications since the 1990s [28]. Since the union of these two techniques many works have been carried out using this method of chromosome analysis. The mallard duck karyotype (*Anas platyrhynchos*, APL, 2n = 80) was hybridized with 57 BAC probes from the *Gallus gallus* library (2n = 78), showing great conservation in the position of most BACs, with two paracentric inversions being found, one in pair 1 (APL1) and another in pair 2 (APL2) [29]. Three species of birds, the Saker falcon (*Falco cherrug*, FCH, 2n = 52), the budgerigar (*Melopsittacus undulatus*, MNU, 2n = 62) and common ostrich (*Struthio camelus*, SCA, 2n = 80) were hybridized with 148 probes from the GGA library that demonstrated a high degree of homeology with GGA [26]. A set of 216 BAC probes (from the GGA and TGU library) was applied in the comparative study between the karyotypes of the domestic pigeon (*Columbia livia*), which has a basal karyotype (2n = 80) and the peregrine falcon (*Falco peregrinus*) with 2n = 50, the result of successive chromosomal fusions. The results showed that the domestic pigeon karyotype is quite conserved, with only one fission event derived from the GGA4 chromosome forming two chromosomes. The peregrine falcon has an extremely modified karyotype with chromosomal fusion rearrangements. In addition, it was possible to establish that breakpoints in birds are more located in regions with a lower concentration of conserved non-coding elements [30].

Thus, it is possible to infer that chromosome painting alone is not capable of demonstrating all the rearrangements that occurred in the differentiation of the karyotypes of avian species. The present study aims to deepen the understanding of chromosomal information in *Charadrius collaris*, a species previously studied by chromosomal painting [18], through the application of BAC-FISH with probes from the GGA and TGU libraries. This is in order to investigate possible rearrangements within of the apparently conserved karyotype of this species and thus better clarify the evolutionary history of the species and the taxon where it is placed.

## Material and methods

### Ethics statement

The Animal Ethics Committee (Comitê de Ética Animal) from Universidade Federal do Pará (UFPA), authorized the present study (Permit 68–2015). JCP has a permanent field permit, number 13248 from “Instituto Chico Mendes de Conservação da Biodiversidade”. The Cytogenetics Laboratory from UFPA has a special permit number 19/2003 from the Ministry of Environment for samples transport and 52/2003 for using the samples for research.

### Samples and chromosomal preparation

The samples used in the present study were the same used in a previous study [18], so no sampling in the wild was carried out this time. Briefly, four specimens of *C*. *collaris* were collected on the island of Otelina (0°45’42.57”S; 46°55’51.86”W, one male and one female) and on Pilão beach (0°47’46.08”46°40’29.64”W, two females), on the coast of Brazil. The samples were collected at six collection points using nets with 12m x 2m and 36mm mesh. The chromosome preparations used in this work were obtained bone marrow technique [31]. Briefly, after the injection of a 0.05% aqueous colchicine solution (0.01 ml per 10 g body weight) and the animal sacrifice by overdose of lidocaine (20mg/ml), the bone marrow was removed from femur and placed in a homogenizer for incubation in a hypotonic solution. The material was fixed with 1 mL of ice-cold Carnoy fixative (methanol and glacial acetic acid in a 3:1 ratio).

### Generation of probes for BAC-FISH

#### Selection of BAC clones

Clone selection was performed following a published protocol [30]. A total of 57 probes from the *Gallus gallus* and *Taeniopygia guttata* libraries were tested, 26 corresponding to macrochromosomes 1–10 of GGA and TGU and 31 to microchromosomes 11–28 of both species (Table 1).

**Table 1:** List of BAC clones used in the work.

	BAC Clone	CCO	GGA/TGU	Status
1	CH261–107E2	1	1	CONSERVED
2	CH261–119K2	1	1	CONSERVED
3	CH261–168O17	1	1	CONSERVED
4	CH261–125F1	1	1	PARACENTRIC INVERSION
5	CH261–184E5	1	1	CONSERVED
6	CH261–18J16	1	1	CONSERVED
7	CH261–25P18	1	1	PERICENTRIC INVERSION
8	CH261–36B5	1	1	PERICENTRIC INVERSION
9	CH261–83O13	1	1	CONSERVED
10	CH261–169E4	2	2	CONSERVED
11	CH261–169N6	2	2	CONSERVED
12	CH261–1J20	2	2	CONSERVED
13	CH261–44D16	2	2	CONSERVED
14	CH261–44H14	2	2	CONSERVED
15	CH261–50C15	2	2	CONSERVED
16	TGMCBA–340P4	2	2	CONSERVED
17	TGMCBA–78C11	2	2	CONSERVED
18	CH261–17B14	3	3	CONSERVED
19	CH261–115J5	3	3	CONSERVED
20	TGMCBA–250J17	3	3	CONSERVED
21	TGMCBA–295P5	3	3	CONSERVED
22	CH261–89P6	4	4	CONSERVED
23	CH261–85H10	4	4	CONSERVED
24	CH261–185L11	4	4	CONSERVED
25	CH261–49F3	6	6	CONSERVED
26	CH261-187M16	7	9	CONSERVED
27	CH261-115G24	MIC	MIC	CONSERVED
28	CH261-71G18	MIC	MIC	CONSERVED
29	CH261-121N21	MIC	MIC	CONSERVED
30	CH261-154H1	MIC	MIC	CONSERVED
31	CH261-60P3	MIC	MIC	CONSERVED
32	CH261-4M5	MIC	MIC	CONSERVED
33	CH261-115I12	MIC	MIC	CONSERVED
34	TGMCBA-321B13	MIC	MIC	CONSERVED
35	CH261-122H14	MIC	MIC	CONSERVED
36	CH261-69D20	MIC	MIC	CONSERVED
37	CH261-90P23	MIC	MIC	CONSERVED
38	TGMCBA-266G23	MIC	MIC	CONSERVED
39	TGMCBA-375I5	MIC	MIC	CONSERVED
40	CH261-42P16	MIC	MIC	CONSERVED
41	CH261-60N6	MIC	MIC	CONSERVED
42	CH261-10F1	MIC	MIC	CONSERVED
43	CH261-50H12	MIC	MIC	CONSERVED
44	TGMCBA-341F20	MIC	MIC	CONSERVED
45	CH261-122K8	MIC	MIC	CONSERVED
46	CH261-40J9	MIC	MIC	CONSERVED
47	CH261-18G17	MIC	MIC	CONSERVED
48	CH261-90K11	MIC	MIC	CONSERVED
49	CH261-103F4	MIC	MIC	CONSERVED
50	CH261-65O4	MIC	MIC	CONSERVED
51	CH261-59C21	MIC	MIC	CONSERVED
52	CH261-127K7	MIC	MIC	CONSERVED
53	CH261-186M13	MIC	MIC	CONSERVED
54	CH261-170L23	MIC	MIC	CONSERVED
55	CH261-28L10	MIC	MIC	CONSERVED
56	CH261-64A15	MIC	MIC	CONSERVED
57	CH261-72A10	MIC	MIC	CONSERVED

#### Preparation of BAC clones for FISH

The BACs were cultured on Luria Bertani Agar (LB Agar) and DNA from the clones were extracted using the QIAprep Spin Miniprep Kit (Qiagen). BACs were labeled by nick translation using Texas red-12-UTP (Invitrogen) and FITC-fluorescein-12-UTP (Roche) prior to purification using the Qiagen Nucleotide Removal Kit [30].

#### Preparation of slides for BAC-FISH

Chromosomal preparations were placed on each half of the slide and allowed to air dry. The slides were washed in 2xSSC (Saline-Sodium Citrate) (Gibco) for 2 min, then they were dehydrated by a series of ethanol washes with concentrations of 70% (v/v), 85% and 100% (2 min at each concentration) and left air drying [29].

#### Fluorescence *in situ* hybridization for BAC-FISH

The probe mixture was prepared by adding 1.5μl of FITC-labeled probe, 1.5μl of Texas Red-labeled probe, 1μl of *Gallus gallus* Hybloc (Applied Genetics Laboratories), 6μl of Hyb I (Cytocell) for one hybridization volume of 10μl and probe concentration of 10ng/μl. Slides were incubated in the hybridization chamber at 37°C for 72 hours and then washed with 2xSSC with 0.05% Tween-20 (Sigma-Aldrich) and stained with DAPI Vectashield Antifade Mounting Medium (Vectorlab) [29].

#### Microscopy

The images were captured using a Carl Zeiss Axio Imager.D2 Microscope, with an Axiocam 503 mono digital camera. The capture process was mediated by the Axiovision 3.1 program (Zeiss). Three different filters were used to acquire images with DAPI, fluorescein isothiocyanate and Texas Red fluorochromes.

## Results

### The karyotype of *Charadrius collaris*

**Fig 1:**
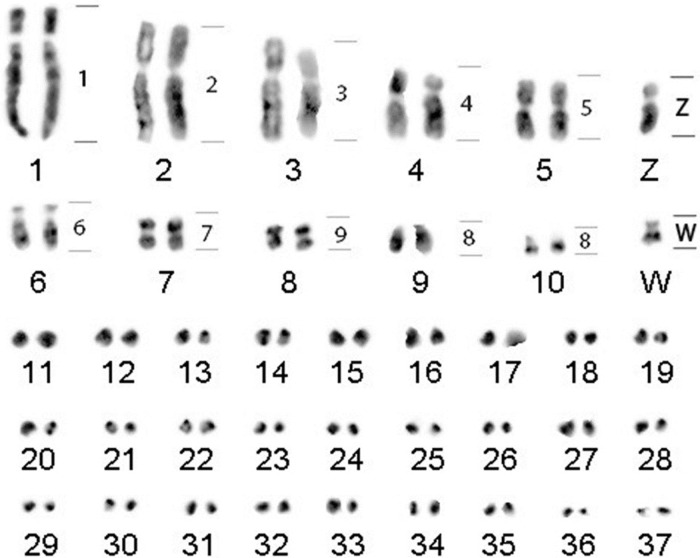
*Charadrius collaris* karyotype showing the location of the whole chromosome probes from *Burhinus oedicnemus* on the right side [18].

**Fig 2:**
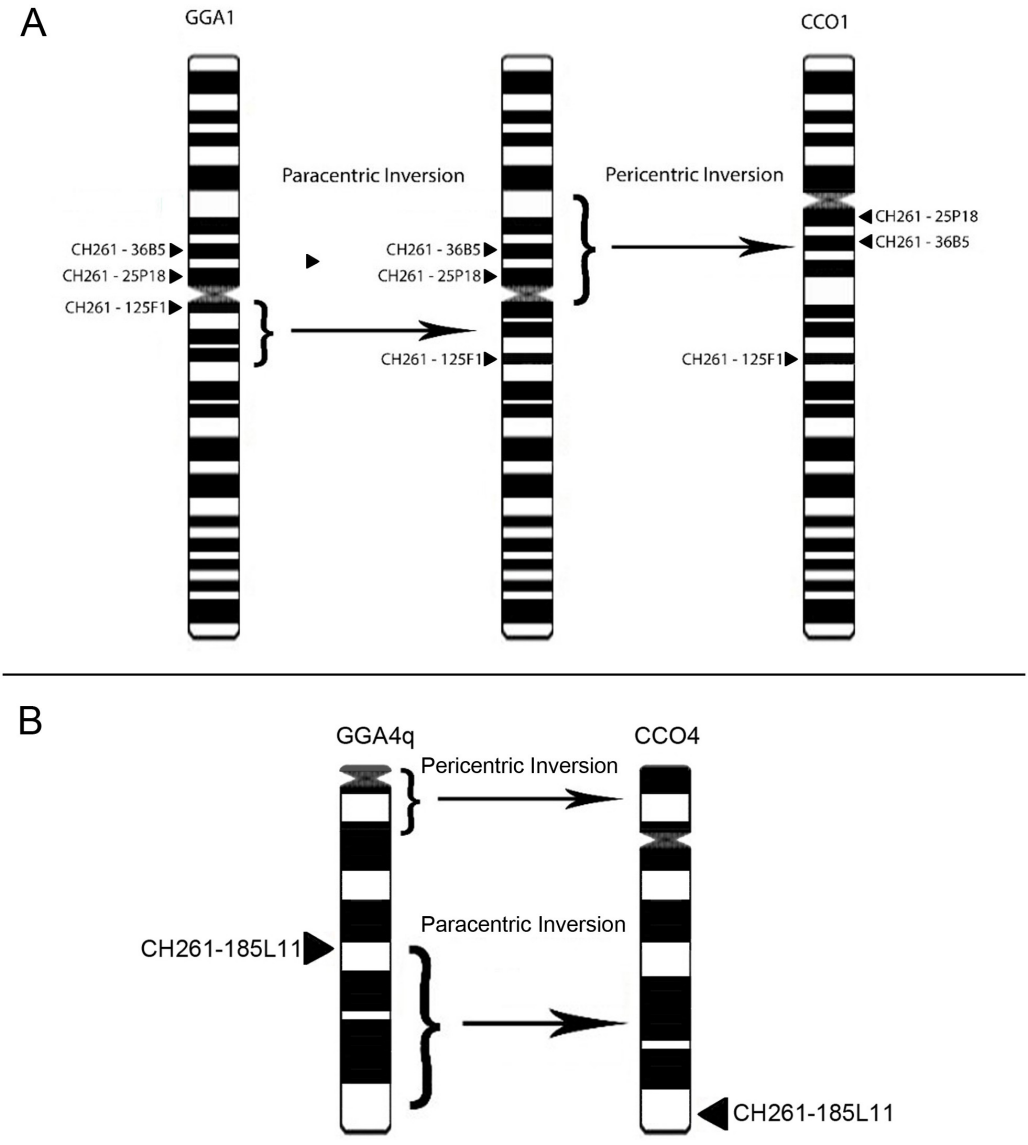
Ideogram containing the positions of the BAC probes in: A) pair 1 of *Gallus gallus* (GGA1, left) and the correspondence of these probes in *Charadrius collaris* (CCO1, right); B) pair 4q of *Gallus gallus* (GGA4q, left) and the correspondence of these probes in *Charadrius collaris* (CCO4, right). *GGA1 = BOE1; GGA4q = BOE4 [8].

As previously described [18], *Charadrius collaris* presented 2n = 76 and FN = 94, with nine pairs of macrochromosomes. Macrochromosome pairs 1 through 8 are bi-armed and pairs 9 and 10 are one-armed. The sex chromosomes (ZW) are one-armed. Fig 1 is a modified version of Fig 2 on the previous publication [18]. In that article, chromosome painting was performed with *Burhinus oedicnemus* (BOE) probes, shown as numbers next to the chromosome pairs.

### BAC—FISH

Comparative mapping of BAC probes from *Gallus gallus* and *Taeniopygia guttata* in *Charadrius collaris* revealed hybridization signal for 26 macrochromosome probes. Probes for microchromosomes 11 to 28 of *Gallus gallus* were also used and revealed 31 hybridization signals.

**Fig 3:**
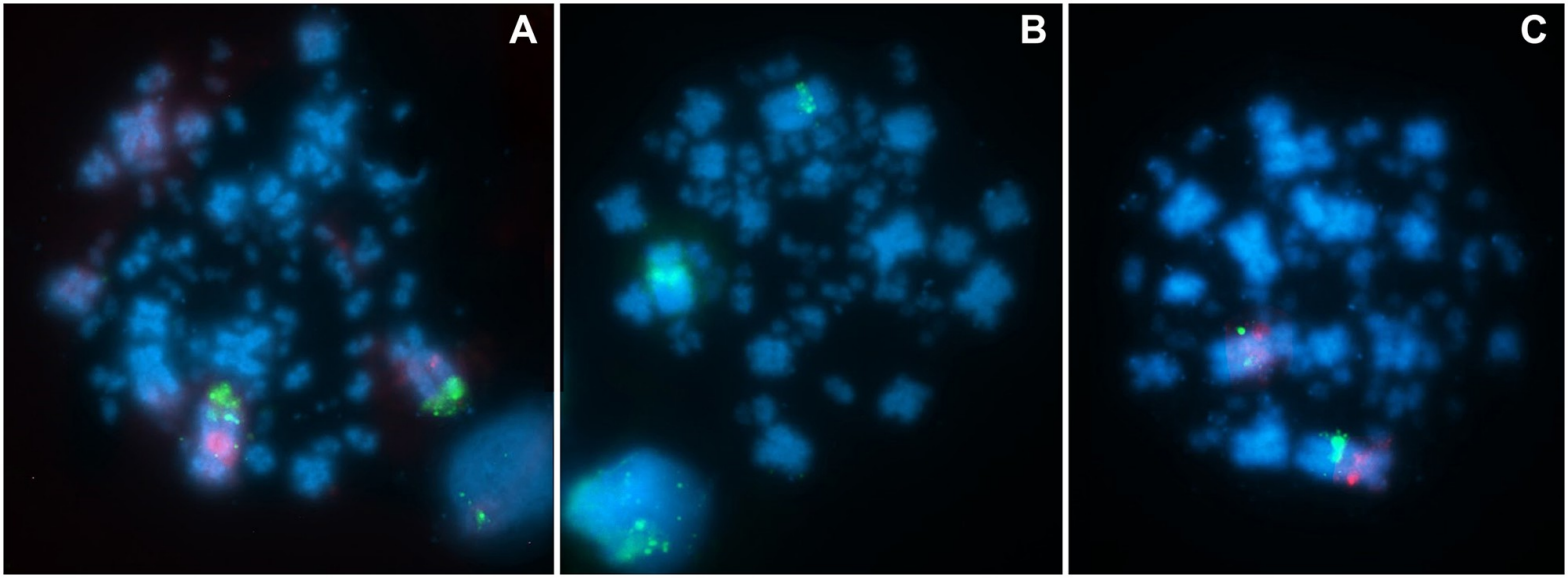
Physical mapping, in *Charadrius collaris* karyotype, of probes A) CH261-125F1 visualized in Texas Red (red) and CH261-184E5 in FITC (green). B) CH261-36B5 in FITC (green). C) CH261-25P18 (Texas Red) and 18J16 (FITC). Chromosomes are stained with DAPI (blue).

Of the 26 macrochromosome probe locations, we found four intrachromosomal differences in relation to the two species (Fig 2), on the probes CH261–125F1, CH261–25P18, and CH261–36B5 (CCO1) and CH261–185L11 (CCO4). All microchromosome probe hybridizations within the CCO karyotype hybridized only on microchromosomes. Examples of the hybridizations are shown below in Fig 3.

Additional images with all the BAC probes hybridized in metaphases of *Charadrius collaris* can be found in the S1 Fig (hybridization to macrochromosomes) and S2 Fig (hybridization to microchromosomes).

## Discussion

The hybridization of the GGA and TGU probes in the CCO karyotype demonstrated intrachromosomal rearrangements in the CCO1, being a paracentric inversion for the CH261–125F1 probe and a pericentric one involving the CH261–36B5 and 25P18 probes (Fig 2A). Another inversion was demonstrated in CCO4, where the CH261–185L11 probe was rearranged by paracentric inversion, from a more central position in the homeologue GGA4q to the telomere region in CCO4 (Fig 2B). This is the first description of these specific inversions. A pericentric inversion was previously described in a chromosome homeologue to GGA1 [32]. However, that inversion happened after a fission that splitted the short and long arms, and the inversion happened in the homeologue of GGA1 long arm while in *Charadrius collaris* this fission never happened. Other inversions were described in birds’ karyotypes [33] but they are different inversions. Finally, a comparison of the morphology of GGA4q and CCO4 shows that a pericentric inversion happened between these two chromosomes. However, at this moment it is not possible to define the direction of this rearrangement.

The comparative analysis of the mapping of the BAC probes of GGA and TGU in the CCO karyotype in relation to those already described in the literature in five other species, *Melopsittacus undulatus* (MUN [25]), *Falco cherrug* (FCH [34]), *Struthio camelus* (SCA [34]), *Falco peregrinus* (FPE [34]), *Falco rusticolus* (FRU [34]) demonstrated a high degree of conservation (Tables 2 and 3). This study did not demonstrate any type of rearrangement between chromosomes, but confirms that the GGA4 chromosome is divided into two chromosomes in *C*. *collaris*, similar to what has been observed in other species [9,18]. This feature is not unique to this species or family. *Burhinus oedicnemus*, despite having a karyotype resulting from several chromosomal fusions that resulted in a decrease in its chromosome number (2n = 40), shares this same characteristic with *C*. *collaris* [8]. Similarly, in the species *Willisornis vidua* (Passeriformes, 2n = 80) the same fission characteristic of chromosome GGA4 [9] occurs. These results reinforce the assumption that the GG4 chromosome is the result of a chromosomal fusion, with separate chromosomes being the form found in the avian ancestral karyotype [3].

**Table 2:** Comparison of chromosomal homeology between 6 bird species—*Charadrius collaris* (CCO), *Melopsittacus undulatus* (MUN), *Struthio camelus* (SCA), *Falco cherrug* (FCH), *Falco pereguinus* (FPE), and *Falco rusticolus* (FRU)—Through of hybridization with BAC probes from macrochromosomes of *Gallus gallus* (GGA). Probes compared according to position in GGA. References: [25, 34; present work].

BAC probes	GGA chromosomes	CCO	MUN	SCA	FCH	FPE	FRU
CH261–107E2	1	1	3	1	3	4	3
CH261–168O17	1	1	3	1	3	4	3
CH261–119K2	1	1	-	1	5	-	-
CH261–25P18	1	1	6	1	5	-	-
CH261–83O13	1	1	3	-	3	4	3
CH261–184E5	1	1	-	-	3	4	3
CH261–125F1	1	1	-	1	-	-	-
CH261–18J16	1	1	-	1	-	-	-
CH261–36B5	1	1	-	1	5	6	5
CH261–169E4	2	2	1	2	2	3	2
CH261–169N6	2	2	1	2	4	5	4
CH261–1J20	2	2	1	2	2	3	2
CH261–44D16	2	2	1	2	2	3	2
CH261–44H14	2	2	1	2	2	3	2
CH261–50C15	2	2	1	2	4	5	4
TGMCBA–340P4	2	2	1	2	2	3	2
TGMCBA–78C11	2	2	1	2	2	3	2
CH261–17B14	3	3	2	3	6	7	6
CH261–115J5	3	3	2	3	12	11	12
TGMCBA–250J17	3	3	2	3	6	7	6
TGMCBA–295P5	3	3	2	3	-	-	-
CH261–85H10	4	4	7	4	1	2	1
CH261–185L11	4	4	7	4	1	2	1
CH261–89P6	4	4	7	4	1	2	1
CH261–49F3	6	6	4	6	9	1	9
CH261–187M16	9	9	5	9	13	12	13
CH261–115G24	10	10	9	10	7	1	7
CH261–71G18	10	10	9	10	7	1	7

**Table 3:** Comparison of chromosomal homeology between 6 bird species—*Charadrius collaris* (CCO), *Melopsittacus undulatus* (MUN), *Struthio camelus* (SCA), *Falco cherrug* (FCH), *Falco pereguinus* (FPE), and *Falco rusticolus* (FRU)—Through of hybridization with BAC probes from microchromosomes of *Gallus gallus* (GGA). Probes compared according to position in GGA. References: [25, 34; present work].

BAC probes	GGA chromosome	CCO	MUN	SCA	FCH	FPE	FRU
CH261–121N21	11	11	-	11	15	14	15
CH261–154H1	11	11	7	11	15	14	15
CH261–60P3	12	12	9	12	4	5	4
CH261–4M5	12	12	9	12	4	5	4
CH261–115I12	13	13	-	13	8	8	8
TGNCBA–321B13	13	13	-	13	8	8	8
CH261–122H14	14	14	8	14	4	5	4
CH261–69D20	14	14	8	14	4	5	4
CH261–90P23	15	15	11	15	1	2	1
TGMCBA–266G23	15	15	11	15	1	2	1
TGMCBA–367I5	17	17	2	17	9	1	9
CH261–42P16	17	17	-	17	9	1	9
CH261–60N6	18	18	12	18	-	-	-
CH261–10F1	19	19	13	19	1	2	1
CH261–50H12	19	19	13	19	1	2	1
TGMCBA-341F20	20	20	10	-	10	9	10
CH261-122K8	21	21	14	21	2	3	2
CH261-40J9	22	22	17	22	18	-	-
CH261-18G17	22	22	17	22	18	-	-
CH261-90K11	23	23	16	23	2	3	2
CH261-103F4	24	24	-	24	16	15	16
CH261-65O4	24	24	15	24	16	15	16
CH261-59C21	25	25	21	25	-	-	-
CH261-127K7	25	25	21	25	-	-	-
CH261-186M13	26	26	18	-	17	16	17
CH261-170L23	26	26	18	26	17	16	17
CH261-28L10	27	27	20	27	19	-	-
CH261-64A15	28	28	-	28	4	5	4
CH261-72A10	28	28	19	28	4	5	4

### Position of BAC probes in *Charadrius collaris* and other bird species

*Gallus gallus* is the most studied bird at the chromosomal level and considered the species with the most similar karyotype to the ancestral bird [25]. *Taeniopygia guttata*, in turn, is the best characterized bird—both at a behavioral, neurobiological and chromosomal level—within the Passeriformes order [35].

The homeologies occurring in CCO, largely in a conserved way, are shared by other species of different orders, but some shared probes do not show the same pattern of arrangement on the same chromosomes, as is the case with the probes shown in Table 2, which were used in the present work. These were also hybridized in the karyotype of three species (*Melopsittacus undulatus*—MUN (2n = 62), *Falco cherrug*—FCH (2n = 52) and *Struthio camelus*—SCA (2n = 80) [25]. Most of differences in the probe locations in the species analyzed in relation to GGA are the result of fission/fusion events and translocations. It is possible to notice that only some probes occurred in different positions in the same chromosomes, indicating the occurrence of inversions, as is the case of the probes that correspond to GGA1, CH261–36B5, 125F1 and 25P18, in CCO1 (Figs 2 and 3). These rearrangements have not been described for any other species so far. An inversion has been reported involving the CH261–125F1 probe, where the BAC-FISH approach is used with GGA probes in *Crotophada ani* (Cucoliformes), demonstrating a pericentric inversion involving two probes in addition to this one [23], unlike what occurs in *C*. *collaris* where the probe CH261–125F1 demonstrates a paracentric inversion.

The conservation of chromosomal position between sequences of chromosomes GGA2 and 3 in *C*. *collaris* was also demonstrated in *Sicalis flaveola* (Passeriformes), where additionally the authors highlight the conservation of the position of probes TGMCBA–340P4, 78C11 and TGMCBA–295P5 and CH261–17B14 [36]. In the present work, the same probes conserved in the homeologous pairs of *C*. *collaris* are demonstrated.

### Conservation on microchromosomes

It is remarkable that there is a very high degree of conservation among microchromosomes in birds, even separated by millions of years of evolution. The microchromosome BAC probes used in the present study demonstrate this degree of conservation in the CCO karyotype, with hybridizations only between the microchromosomes (Table 3), as well as in SCA, using the same set of probes used in the present work [25].

Different from the results for SCA, in MUN and FCH many rearrangements involving microchromosomes were observed, such as the probe TGMCBA–367I5, which corresponds to GGA17 and is found rearranged in chromosomes MUN2 and FCH9 [25]. Many rearrangements involving macro and microchromosomes were demonstrated, but there were no significant changes between the FCH and FRU karyotypes, with many differences between the FCH/FRU and FPE karyotypes [29]. *Charadrius collaris* maintains its diploid number close to that of the ancestral avian karyotype (2n = 80), which is very similar to the GGA karyotype (2n = 78), maintaining the chromosomal conformation between the microchromosomes of the two species.

### The karyotype of *Charadrius collaris*

**Fig 4:**
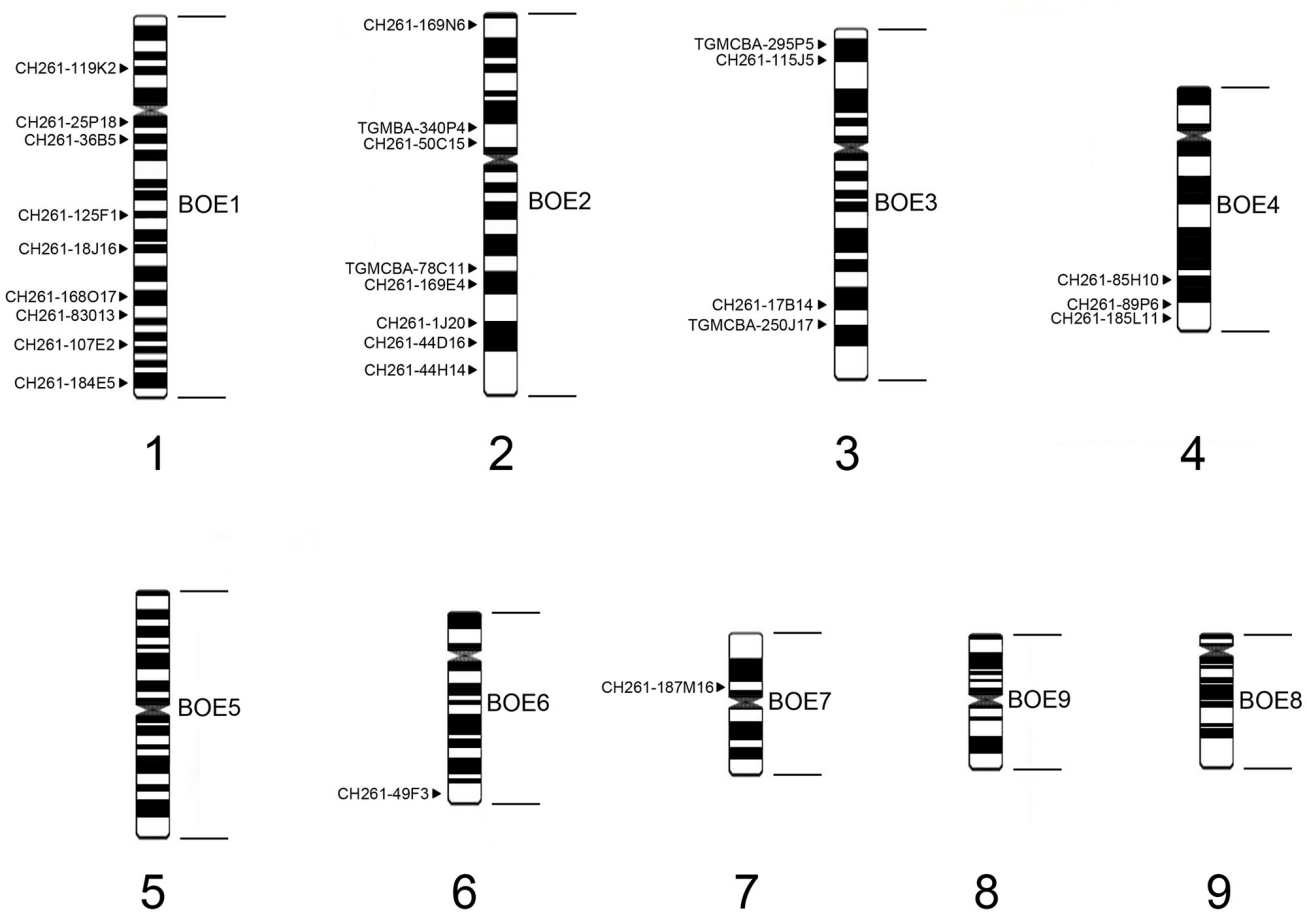
A comparison of the mapping of whole chromosome probes (right side) and BAC probes used in this work (left side) on the idiogram of chromosome pairs 1–9 from *Charadrius collaris*.

Only recently did *Charadrius collaris* have its first karyotype description by classical and molecular cytogenetics with whole chromosome probes [18], in which the 2n and chromosomal morphology of CCO was demonstrated. That work demonstrated homologies between the chromosomes CCO1 (BOE1), CCO2 (BOE2), CCO3 (BOE3) CCO4 (BOE4), CCO5 (BOE5), CCO6 (BOE6), CCO7 (BOE7) and CCO8 (BOE9). These same previously mentioned probes marked the CCO W chromosome. In the present work, we were able to associate the data from the CCO karyotype mapping from the whole chromosome probes [18], with the BAC-FISH mapping with probes from the GGA and TGU libraries (Fig 4). It was possible to demonstrate the presence of rearrangements in CCO1, which previously could not be detected using whole chromosome probes.

## Conclusion

We described here for the first time the chromosomal mapping using BAC probes of the *Gallus gallus* and *Taeniopygia guttata* libraries in the karyotype of *Charadrius collaris*, which revealed an apparent conservation in the karyotype. *Charadrius collaris* showed the separation of GGA4 into two chromosomes, a feature that is very common in other avian orders, supporting the assumption that the configuration with separate chromosomes is the ancestral form. We suggest that this may be a feature common to the genus *Charadrius*, due to the high conservation of 2n and chromosomal morphology between the species.

We found three chromosomal inversions (pairs CCO1 and CCO4) for this species compared to *Gallus gallus* and *Taeniopygia guttata* that were only possible to detect using the BAC-FISH technique. Further studies are needed to improve the understanding of chromosomal evolution within the order Charadriiformes and thus clarify whether these characteristics demonstrated here are specific traits of *Charadrius collaris* or whether other species share these characteristics.

## Supporting information

S1 Fig Metaphases of *Charadrius collaris* showing the hybridization to macrochromosomes of BAC Clones probes from *Gallus gallus* and *Taeniopygia guttata*. (TIF)

S2 Fig Metaphases of *Charadrius collaris* showing the hybridization to microchromosomes of BAC Clones probes from *Gallus gallus* and *Taeniopygia guttata*. (TIF)
