# Supplementary figures and images for: A conserved karyotype? Chromosomal rearrangements in Charadrius collaris detected by BAC-FISH
Source: PLoS One. 2023 Jan 11;18(1):e0280164. doi: 10.1371/journal.pone.0280164 (PMC9833595; doi:10.1371/journal.pone.0280164)

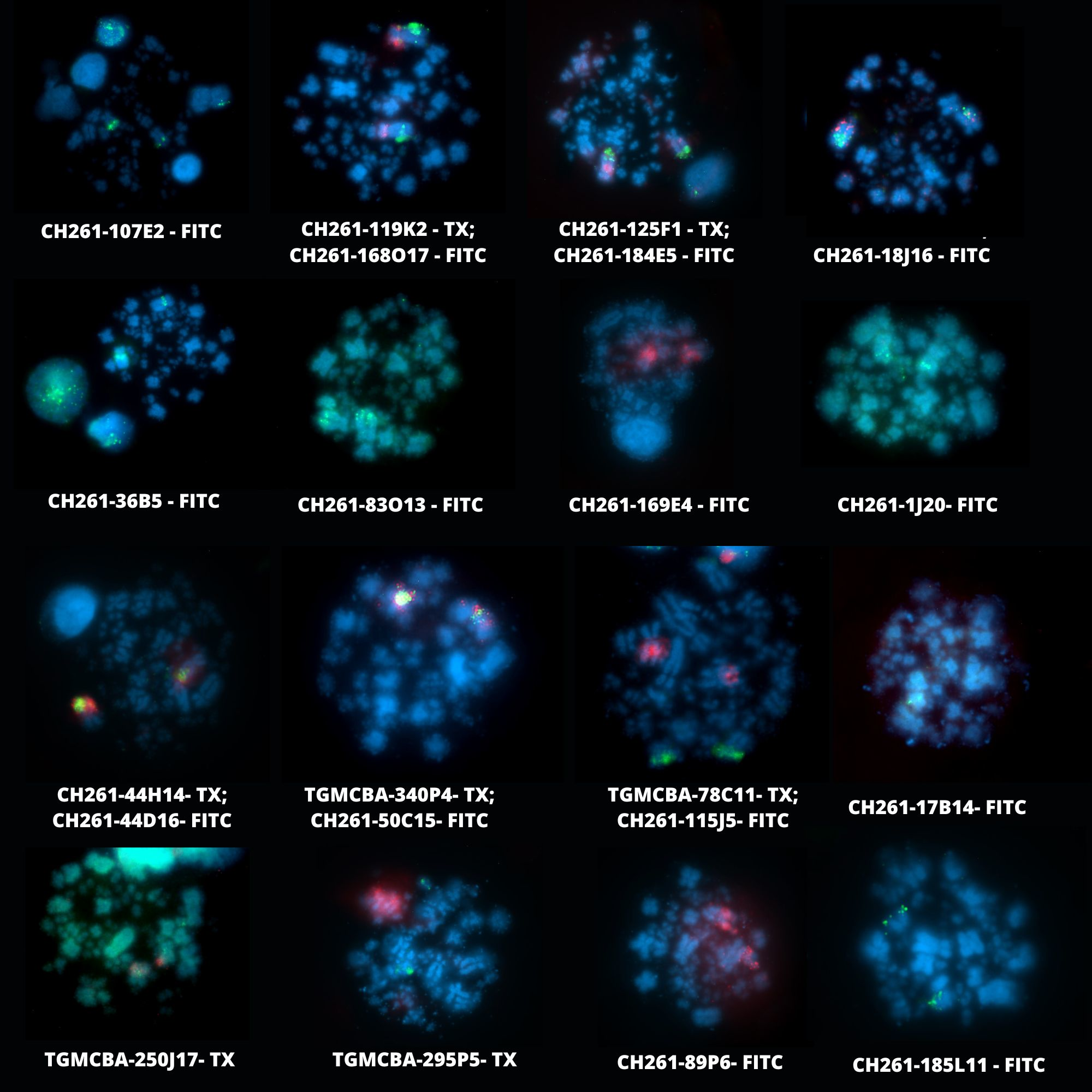

Supplement: S1 Fig — (TIF) [file pone.0280164.s001.tif]

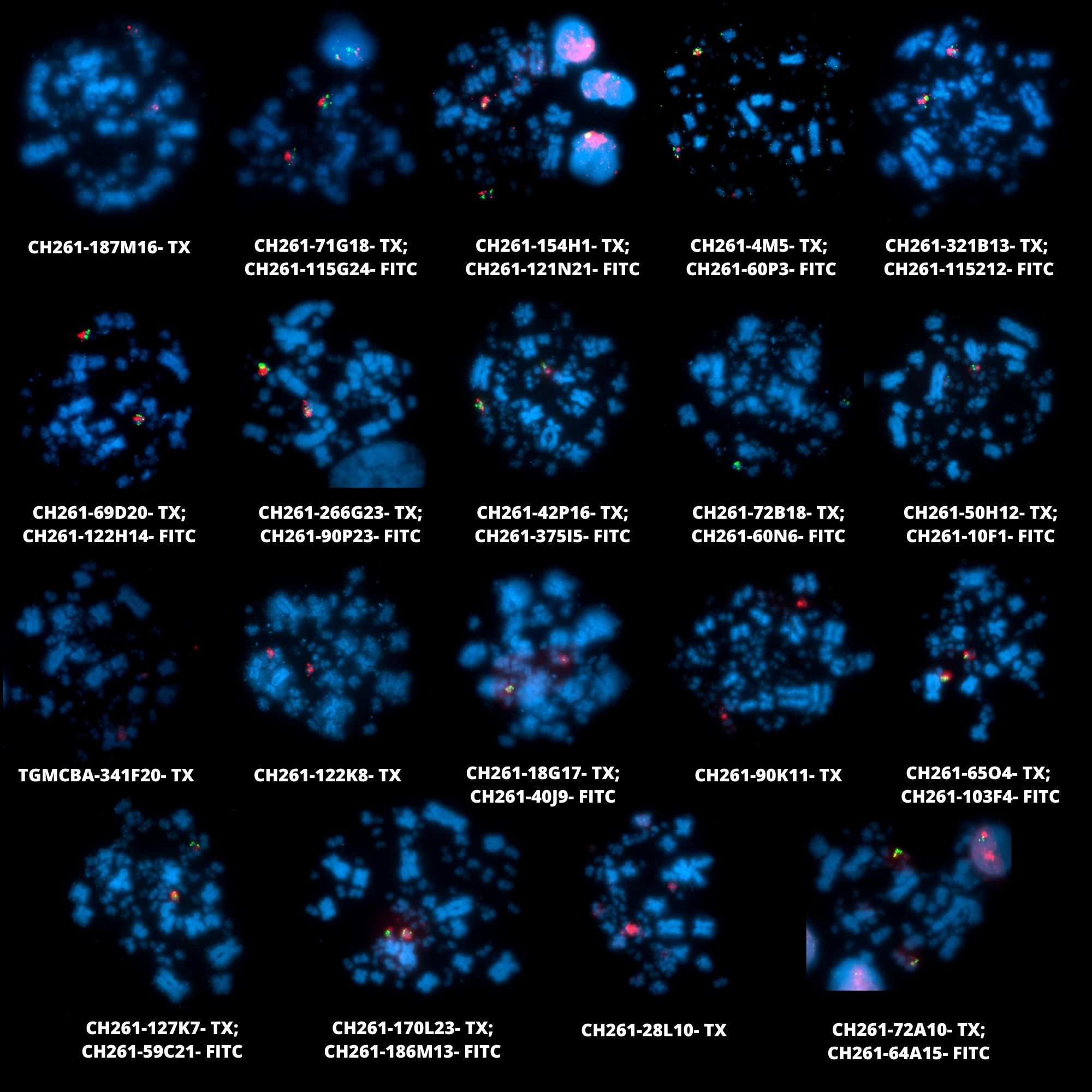

Supplement: S2 Fig — (TIF) [file pone.0280164.s002.tif]
